# Social Prescribing and Lifestyle Medicine—A Remedy to Chronic Health Problems?

**DOI:** 10.3390/ijerph181910096

**Published:** 2021-09-26

**Authors:** Alicja Baska, Donata Kurpas, Joyce Kenkre, Josep Vidal-Alaball, Ferdinando Petrazzuoli, Miriam Dolan, Daniel Śliż, Joanne Robins

**Affiliations:** 1 Department of Lifestyle Medicine, School of Public Health, Centre of Postgraduate Medical Education, 01-826 Warsaw, Poland; dsliz@cmkp.edu.pl; 2 Department of Family Medicine, Wroclaw Medical University, 51-141 Wrocław, Poland; donata.kurpas@umed.wroc.pl; 3 The European Rural and Isolated Practitioners Association (EURIPA), 92200 Nuilly-sur-Seine, France; ferdinando.petrazzuoli@gmail.com; 4 Faculty of Life Sciences and Education, University of South Wales, Pontypridd CF37 1DL, UK; joyce.kenkre@southwales.ac.uk; 5 Health Promotion in Rural Areas Research Group, Gerència Territorial de la Catalunya Central, Institut Català de la Salut, Unitat de Suport a la Recerca de la Catalunya Central, 08272 Sant Fruitós de Bages, Spain; jvidal.cc.ics@gencat.cat; 6 Fundacio Institut, Universitari per a la Recerca a l’Atencio Primaria de Salut Jordi Gol i Gurina, 08272 Sant Fruitós de Bages, Spain; 7 Faculty of Medicine, University of Vic-Central University of Catalonia, 08500 Vic, Spain; 8 SNAMID (National Society of Medical Education in General Practice), 81100 Caserta, Italy; 9 Center for Primary Health Care Research, Department of Clinical Sciences, Lund University, SE-221 00 Lund, Sweden; 10 Maple Healthcare, Lisnaskea, Enniskillen BT92 0GT, UK; miriamdolan@btinternet.com; 11 Royal College of General Practitioners, Belfast BT7 2JD, Northern Ireland, UK; 12 Polish Society of Lifestyle Medicine, 00-388 Warsaw, Poland; 13 Shropshire Council, Midlands NHS England, Shrewsbury SY2 6ND, UK; jo.robins@shropshire.gov.uk

**Keywords:** social prescribing, lifestyle medicine, public health, family medicine, non-communicable diseases, social determinants of health

## Abstract

Social prescribing has been identified as a chance to take a holistic approach to people’s health and wellbeing, especially for people with one or more long-term conditions. Its systemic implementation was a part of the recent United Kingdom National Health Service Long Term Plan. With a lifestyle medicine focus on equipping patients in tools necessary for self-care and self-management of their lifestyle-related health problems that coexists with the need for creating an environment supporting healthy choices, a social prescribing model seems to offer a promising strategy for advancing lifestyle medicine. This idea was discussed during a meeting hosted by the Polish Society of Lifestyle Medicine in collaboration with European Rural and Isolated Practitioners Association, Polish Society of Young Family Doctors (“Młodzi Lekarze Rodzinni”), British Society of Lifestyle Medicine and European Lifestyle Medicine Council in June 2020. The aftermath—this position statement is an Authors’ attempt at summarizing the common ground for social prescribing and lifestyle medicine. It collects experiences of practitioners and researchers from five European countries as well as making recommendations for applying this model in Poland. Despite referring to local conditions, it might provide universal takeaway messages for any healthcare providers interested in combining social prescribing with lifestyle medicine practice.

## 1. Social Prescribing and Lifestyle Medicine—A Natural Alliance?

This opinion paper was written in the aftermath of an online meeting [1] hosted by the Polish Society of Lifestyle Medicine (PSLM) in collaboration with the European Rural and Isolated Practitioners Association (EURIPA), the Polish Society of Young Family Doctors (“Młodzi Lekarze Rodzinni”), the British Society of Lifestyle Medicine (BSLM) and the European Lifestyle Medicine Council (ELMC) in June 2020.

In January 2019, the United Kingdom (UK) National Health Service (NHS) issued its Long Term Plan and the Universal Personalised Care Plan, presenting social prescribing (SP) as a key component, “taking a holistic approach to people’s health and wellbeing” [2]. SP was described as being designed to “work for people with one or more long-term conditions, who need support with their mental health, who are lonely or isolated and/or who have complex social needs which affect their wellbeing”. In the context of an epidemiological situation in the UK and an analogical situation in most of the developed countries around the world, it is clear that a vast majority of patients who fulfil the above mentioned prerequisites to become SP beneficiaries would also fit into a target group for lifestyle medicine (LM) interventions. To unleash the full potential of LM and SP, the quoted definition could be further extended for SP to work not only with “people with one or more long-term conditions”, but with patients having risk factors for those—such as elevated cholesterol or blood glucose levels, etc.

A sustainable lifestyle change that could directly address the underlying causes (rather than treat only the superficial symptoms) of the most challenging chronic health problems is a long process. Some studies estimate that, despite a great potential and proven effectiveness of lifestyle modification for disease prevention, management and even reversal, the non-adherence rates to lifestyle counselling can be as high as 70% [3]. Undoubtedly, there are many factors that contribute to these unsatisfactory results—both on the part of healthcare providers (deficiency of adequate training in the area of lifestyle medicine, not enough time for effective lifestyle intervention during primary care visits, lack of reimbursement) as well as on the part of patients (for example, insufficient knowledge and low level of health literacy, low socioeconomic status). There is a growing body of evidence indicating that friends, family and social support can act as facilitators to lifestyle behaviour change and play a critical role for example in the prevention of long-term excess weight gain. The importance of approaches that address the interconnected influences of levels of individual, peer/family group, health care team, neighbourhood resources and cultural context have also been particularly emphasized in low-resources settings, disproportionately affected by lifestyle-related diseases. These approaches are said to increase the likelihood that patients could sustain behaviour changes outside the healthcare setting [4].

Lifestyle medicine focuses on empowering patients and equipping them with tools necessary for self-care and self-management of lifestyle-related health problems as well as aims to create an environment supporting a healthy lifestyle and healthy choices. Thus, a social prescribing model seems to offer a wide range of solutions to address some of the above-mentioned barriers to lifestyle modification adherence.

It is worth articulating that the concept of SP had been functioning under different names and formats in various countries long before it became the apple of NHS’s eye and was introduced on a systemic level, within a structured framework. The principles of social prescribing had been increasingly applied to older populations, in order to deal with loneliness, improving levels of physical activity and mental well-being [5].

The next sections of the article present a general overview of the SP as well as the collective experiences of practitioners and researchers from five European countries: United Kingdom (England, Wales and Northern Ireland), Catalonia and Italy. We also define the need and circumstances requiring potential application of a social prescribing model as a tool for lifestyle medicine advancement that, despite referring directly to local conditions in Poland, might provide some universal takeaways for any healthcare provider interested in combining social prescribing with lifestyle medicine practice.

## 2. Social Prescribing—Key Principles

As pointed out by Islam [6], social prescribing, sometimes referred to as “community referral”, does not seem to have a one agreed definition. Different entities responsible for SP provision in UK (where the term originated) propose few explanations of the concept that could be jointly summarised as “a non-medical referral option for GPs, for other medical and some non-medical professionals and also for self-referral to the sources of support”. 

In England—a world pioneer in implementing social prescribing at the healthcare system level—the recently published NHS Long Term Plan includes social prescribing as an acknowledged service provision with the new workforce designed to support primary care, offering a non-clinical intervention through a trained Link Worker. Implementing SP into the healthcare system in this way allowed for defining it as a way of enabling GPs (General Practitioners) and other frontline health workers to refer patients to a Link Worker (LK). LK offers a face-to-face conversation during which a personalised solution is co-designed to enable people to access local initiatives and projects provided by the voluntary, community, or social enterprise sector. The patient therefore works out what will work for them, which may be educationally, culturally, employment or environmentally driven (examples would include befriending groups, information on debt, access to employment support, participation in a physical activity or nutrition class, access to arts classes, walking groups, weight management sessions, music therapy and many others). 

Social prescribing recognizes the importance and relevance of the impact of wider determinants of health, which cannot be treated by a clinical approach alone. This approach to healthcare has a huge potential to achieve results, which go beyond medicine, and can work alongside clinical interventions in a transformative way by engaging people in new conversations which improves their wellbeing and health. Social prescribing can be utilised to support lifestyle change, reach out to people who are lonely and isolated and address unmet needs which can exacerbate long term health conditions. In England, as in many other countries across Europe, health and care services are facing unprecedented demand. In the UK, it is reported that around 30% of patients visit their GP for a social reason [7].

People often experience co-morbidities and social issues at the same time, and may need care from different providers simultaneously. Integrated systems can be designed to organise treatment and prevention so that (health and non-health) services are better coordinated across the whole range of care. Addressing the challenges posed by chronic diseases also requires a change from centralised (typically large central acute systems) to decentralised services (increased primary care facilities in communities). 

It is important that building of the evidence base continues on a range of levels, including the impact on patient’s health and well-being. The impact of the one-to-one conversation between the Link Worker and the patient, the impact of the ‘social prescriptions’ accessed in the community and the impact on primary care. Current evidence is focused on the impact of the model on primary care, the acute sector, but there is limited evidence on the impact of the model in mental health services; however, the approach has the potential to reach beyond health sectors. 

## 3. Different Faces of Social Prescribing in Europe

The different schemes and stages of SP implementation across Europe seem to reflect the multitude of SP definitions mentioned in the previous paragraph.

### 3.1. United Kingdom

#### 3.1.1. England

In England, the social prescribing model described by the NHS England Long Term plan and supported by guidance issued on what constitutes good practice [2] consists of a number of key components such as: a referral mechanism, assessment of needs, through the one to one conversation with the Link Worker, development of a personalised plan, identification of the social prescriptions needed, access to the social prescriptions, follow up contact with the Link Worker, and recording of the impact and outcomes on the patient. In this way, the patient’s needs are addressed in a more holistic way. Additional guidance has been provided to primary care providers illustrating broader principles for the delivery of a system wide model of success including partnership working with the local government, VCS and community, workforce development and support for community groups. Other essential ingredients include governance, use of population health data to assess need, data collection on outcomes and commitment to funding. This is the biggest investment in social prescribing by any national health system so far and legitimises community-based activities and support alongside medical treatment as part of personalised care [2].

#### 3.1.2. Wales

The Well-being of Future Generations (Wales) Act (2015) [8] is a document setting out the ambition, permission and legal obligation to improve social, cultural, environmental and economic well-being in Wales. It was envisaged that the Act would make organisations think more about the long-term outcomes, to work better with people and communities and each other, to prevent problems and improve access to wellbeing services and activities and provide a more joined up approach. Thus, it also created new opportunities for SP implementation. 

The Welsh Government in Taking Wales Forward 2016–2021 has an acknowledged commitment to social prescribing in the context of sectors (e.g., health, social care, social groups, charities, volunteer groups, housing) working together for the benefit of the community (WG 2016a). This was also supported by the Welsh NHS Confederation in acknowledging that social prescribing models seek to enable people and communities to come together for positive change (WNC 2017). The Welsh Government is also committed to the support for research and creating the evidence base for practice in primary care. In 2008, it funded the Wales School of Primary Care Research which has been continued to be funded and is now known as PRIME (Primary and Emergency Care Research Centre) (WG 2016b). Even though PRIME has distinct clinical research areas, there were always cross cutting themes of lay involvement and engagement, social care and the third sector, communication and knowledge mobilisation (PRIME 2020).

In the last funding round for government support for the research infrastructure in Wales PRIME gained funding for a further five years, but, within that, extra funding was granted for the Wales School of Social Prescribing Research (WSSPR 2020). WSSPR was established to develop social prescribing evaluation methodology so as to enable the provision of evidence and quality standards of evaluation. It is a virtual all-Wales school, building on the work previously completed by the Wales Social Prescribing Research Network (WSPRN) (WCVA 2020). The Welsh Government office of Health & Care Research Wales has provided infrastructure funding for the development of: Methodological framework/guidance; Training resources for researchers: How to evaluate social prescribing; lessons log for practice evaluators. There is also provision for the maintenance of Wales Social Prescribing Research Network (WSPRN) with over 300 members.

#### 3.1.3. Northern Ireland

Social Prescribing in Northern Ireland is primarily delivered by SPRING Social Prescribing [9] following on from a pilot project delivered by Bogside and Brandywell Health Forum (BBHF) in the Derry/Londonderry region between 2015 and 2018. SPRING Social Northern Ireland’s Healthy Living Centre Alliance (HLCA) and Scottish Communities for Health and Wellbeing (SCHW) have now created a partnership. The funding comes from the National Lottery, the Department of Agriculture, Environment and Rural Affairs and the Housing Executive. People are identified and referred by their primary health care professional using a specific digital platform [10]. mPower [11] is another social prescribing project which will run from 2017 to 2021 and is supported by the European Union’s INTERREG VA Programme. The project has created a cross-border service for older people (age 65+) living with long-term conditions across the Republic of Ireland, Northern Ireland and Scotland.

Both programmes employ Community Navigators/Link Workers to work with people referred from health and care services to develop wellbeing plans, linking them to activities in their community and at times connecting them to technology to enhance support for health and wellbeing. The aim is to empower patients and communities, support greater independence, reduce reliance on primary healthcare, and ultimately deliver better health outcomes for people and society. The main challenges are the sustainable funding of both organisations going into the future as well as establishing a culture shift from the medical model to the social model of health among their communities and health care professionals.

Miriam stated that, from personal experience as a GP principal working in the largest rural practice in Northern Ireland [12], as a member of the local Integrated Care Partnership and regional Local Medical Council and as a practicing social farmer [13], it cannot be stressed enough that successful social prescribing initiatives depend on strong local relationships. This is between the multiple sectors including the Community and Voluntary, Primary Care, Secondary Care, Social and Domiciliary Care, public health and importantly local government/council. She felt it was important to fully appreciate each other’s role in promoting recovery, rehabilitation, primary and secondary prevention in and of our communities, and we need to challenge each other’s and our own perceptions. Through collaboration rather than cooperation, we break the well-meaning silos that can prevent desired outcomes. The identification and supporting of our local champions are essential to make social prescribing work especially in the initial phase. 

### 3.2. Catalonia

Social prescription in Catalonia was first introduced as a pilot program in 2012. One of its main characteristics is that it was developed specifically from primary health care and uses the Health Assets model [14]. 

The program has a website for the introduction of activities and community resources in each territory [15]. Social prescription is incorporated through a specific module into primary care electronic medical notes. The module consists of a link to the asset finder that allows, with just one click, to access the activities near the primary care centre depending on the sex and age of the person. The module also consists of two questionnaires for the assessment of emotional well-being (Warwick Edinburgh Mental Well-being Scale- SWEMWBS) and the OSLO-3 social support questionnaire. The module allows the practitioner to write down the referral and to give to the patient a social prescription document with information and contact details of the activity [16]. 

For the implementation of the program of social prescription in a territory, city councils, public health services and primary centres are necessary to develop the following phases of implementation: evaluation of the implantation of the program and creation of alliances, creation of a working group, training of the primary care leaders, identification of community assets, training of other primary care professionals and to start the evaluation of the program.

At the Primary Care level, an action protocol is established that consists of five steps: detection, advice/motivation, referral and monitoring and evaluation of patients. Screening includes identifying potential candidates among people being visited and verifying the need for help with the goal of improving their socialization through a community activity. Motivation counselling is completed taking into account the socioeconomic and family context of the individual. This is why it is necessary to establish a climate of trust and apply an appropriate communication style. The use of the motivational interviewing is recommended. The program proposes a follow-up of people who receive a social prescription at 15 days and at 2 and 6 months through an evaluation of well-being and social support [16].

Two leaders from the primary care teams or local authorities interested in implementing social prescription are identified. These leaders will receive training and material to facilitate the initial introductory tasks to create and train a local working group.

Since the start of the project, 288 professionals (97 in public health and 191 in primary care) have received training on social prescription. Twenty-four meetings have been held with local councils and entities at the local level for the dissemination of the program. In January 2018, 433 health centres, 842 libraries and 176 city councils were incorporated into the health assets website. In total, in 2017, the website had 344 activities and 1622 resources. A total of 5817 visits were made to the website between 2015 and 2017 [17].

### 3.3. Italy

For the time being, social prescribing has not been organized, evaluated or brought under a broad definition of Social Prescribing practice in Italy. What has been observed is a rise of initiatives that could be described as having SP components, mostly delivered by the voluntary and community sector organisations.

A representation of such initiatives—mostly dedicated to the elderly—are initiatives managed by so-called “Social circles” [18]—a sort of community centres that involve patients in organised socialising activities like playing cards, dancing, music, theatre, walking tours of the city, etc. It is worth noting that their distribution across the country is not homogenous. There are significant urban-rural divides and North–South disparities in the access to services of that type—typically observed in Italy also in other sectors

The community centres are public places where members of a community tend to come together for group activities, social support, public information and other purposes. They are open for the whole community or for a specialized group within the larger community. Some of them are religious in nature, affiliated and ran by the Italian Christian community.

Apart from everyday activities, community centres also offer a few organized day retreats. Although these are not well-structured, long-term, planned and monitored interventions, they do have a therapeutic potential for addressing the problem of loneliness among the beneficiaries of these activities. In the future, they could provide a promising starting point for SP development in Italy and collaboration between healthcare system, healthcare providers and communities. 

The examples presented above (and during the online meeting that preceded creating this opinion paper) seem to serve as a good illustration of the NHS England model of social prescribing [2] in action. Its key elements “that need to be in place for effective social prescribing” include:

- easy referral from all local agencies;
- workforce development;
- common outcomes framework;
- support for community groups;
- collaborative commissioning and partnership working;
- creation of a personalized plan.

It can be concluded that the countries in which SP implementation seem to be more advanced (as for the time being) indeed include more of the listed key elements. The case studies show also that the common challenge in SP implementation is the funding (and its sustainability—more threatened if the responsibility for the SP funding lies heavily on the third sector rather than organized at the healthcare system level). 

## 4. Social Prescribing and Lifestyle Medicine in Poland—In Need for New Solutions for the Broken Healthcare System

As presented in the Introduction, social prescribing could be perceived as a promising tool for lifestyle medicine advancement, extending the impact range of a conventional model of a healthcare system. This novel approach drew the attention of the originators of this opinion statement for two main reasons. One of them is a lack of satisfactory possibilities to practice effective lifestyle medicine interventions within a conventionally structured public healthcare system (with the changes needed both at undergraduate and postgraduate medical education level as well as within the organization of the health care services delivery). The second one—the awareness of the drivers of health behaviours that require us to look at an individual patient much more broadly, including the environment they live in—is an ally or an obstacle in the pursuit of health.

Even though the major threats for the health of European citizens remain similar among most of the European countries, each country mentioned in the article differs in the way their healthcare systems operate to address them. Since the purpose of the meeting that resulted in creating this particular opinion statement was to open a discussion on social prescribing in Poland, it is worth depicting the specific circumstances that make this approach particularly needed and promising in this country. 

The prevalence of lifestyle-related diseases in Poland, as well as the distribution of the underlying risk factors has been generally increasing over past decades, with only a few small exceptions, for example smoking rates. The latest edition of the report by the National Institute of Public Health-National Institute of Hygiene (NIPH-NIH): “Health status of Polish population and its determinants 2020” [19] showed that behavioural risk factors are responsible for nearly 44% of deaths, and account for almost 36% of disability-adjusted life years in Poland. As highlighted in the report, behavioural risk factors contributed to a higher proportion of deaths from cardiovascular diseases or cancer and in all-cause mortality in Poland more than the average from all the other countries analysed in the most recent Global Burden of Disease Study from 2019 (respectively 52% vs. 49%, 42% vs. 37% and 44% vs. 38%). The most significant risk factors include hypertension accounting for over 22% of deaths in Poland, tobacco use (20%) and unhealthy diet (20%). The percentage of individuals, who were overweight or obese, was found to be over 60% of Polish population. This clearly indicates the urgent need for implementing systemic solutions at an individual level as well as a whole societal level in order to transform the obesogenic environments currently being experienced by the population. 

Taking into account the status quo, there is no doubt that such transformation would require dramatic changes in the provision of the health services in the future. Poland like many countries relied on inpatient secondary care. The excessive use of hospital care coupled with poor financial management has resulted in a common occurrence of hospital financial deficits [20]. It is also worth noting that the total health spending—both as a share of GDP as well as per capita—remain amongst the lowest in the European Union. The allocation of health expenditures within the EU appears to differ. According to 2018 data presented by the European Statistical Office [Eurostat], on average in the EU, public and private expenditure on preventive care accounted for 2.8% of total health expenditure compared to 2.3% in Poland [21]. Similar data were collected in an audit during 2012–2015 which was led by the Polish Supreme Audit Office. The results and recommendations presented in the report published in 2018 “Healthcare system in Poland—current state and desirable direction of change” state that financing of prevention was both lacking and wrongly allocated [20]. Even greater disparity can be observed when comparing the preventive care expenditure in relation to population size. The EU average is over four times greater for preventive care spending than in Poland (82 vs. 19 € per inhabitant) [21]. On the other hand, recent years were marked with a growing interest in public discussion on prevention as well as a continuing search for solutions to manage NCDs with non-pharmaceutical interventions, also at the healthcare system level (some examples include recently launched programs: POZ Plus [22] focused on prevention in the biopsychosocial model and Prophylaxis 40 Plus (lab tests, blood pressure, body weight, height, waist circumference, BMI, regularity of the heart rhythm) program directed to all aged 40 and over). This could possibly create a window of opportunity for the LM/SP advancement. 

Another challenge faced by the Polish healthcare system is the shortage of healthcare professionals. The numbers of doctors and nurses are the lowest in the EU (2.4 and 5.1 per 1000 of population, respectively). The percentage of general practitioners (GPs) is the second lowest in the EU (9% vs. EU average: 23%). To compensate for this deficit, paediatricians and internal medicine specialists are allowed to work as GPs. Medical personnel are also unevenly distributed—the density of doctors varies by almost 70% from district to district [23]. Insufficient outpatient care, poor financial management and shortages in the healthcare workforce have resulted in limited access to health services for general population, long queues and shortened visit times serve as examples of their consequences. They also add up to the burden of other obstacles in lifestyle medicine provision, mentioned in the Introduction, collectively leading to failures in addressing behavioural risk factors. 

All of the above emphasize the need for re-organization of the current structure of healthcare system, justifying the need for seeking and developing new tools as well as methods that could help in addressing some of the most pressing issues. The international experience from SP implementation and development presented in the previous section, juxtaposed with the specific data regarding the situation in Poland, could serve as an initial guidance to include SP in the discussion on the healthcare reform and lifestyle medicine advancement. 

## 5. Conclusions

As outlined in the report “Social prescribing in Wales” [24], “social prescribing is an opportunity to implement sustained structural change to how a person moves between professional sectors and into their community. Social prescribing is part of a wider movement that signifies a shift from traditional top-down models of care delivered in hospitals and GP surgeries to a non-medical, more networked approach by placing the patient at the centre of their care, promoting independence and personal responsibility, and contributing to the common good.” When lifestyle change and health behaviours are discussed, SP has the potential to bridge the gap between the knowledge (awareness of health risks and advised—by healthcare professionals—lifestyle modification) and behaviour (adapting and sustaining a new lifestyle). It also provides an opportunity for extending a multidisciplinary care over the patient—one of the paradigms of successful lifestyle medicine interventions. Although the analysis of effectiveness of social prescribing interventions exceeds the framework of this article, the Authors want to emphasize the need for proper planning and delivering SP programs. The available body of evidence allows for presenting SP as a tool with great potential for reducing lifestyle-related burden although requires from prospect SP implementors reviewing good practices and acknowledging programs that failed to deliver any notable change in health outcomes of its beneficiaries. The Authors also believe that future discussions on lifestyle medicine provision via social prescribing should focus next on identifying local (national) stakeholders and assessment of the opportunities for the system-level implementation, considering sustainable financing schemes as well as should include a more thorough analysis that would compare the different approaches to tackle the deficiencies in the healthcare system.
